# Greater anterior pelvic tilt and lumbar mobility in females compared to males undergoing periacetabular osteotomy: A matched cohort study

**DOI:** 10.1002/jeo2.70167

**Published:** 2025-02-10

**Authors:** Maximilian Fischer, Lars Nonnenmacher, Andreas Nitsch, Matthias R. Mühler, Andre Hofer, Georgi I. Wassilew

**Affiliations:** ^1^ Center for Orthopaedics, Trauma Surgery and Rehabilitation Medicine University Medicine Greifswald Greifswald Germany; ^2^ Department of Radiology and Neuroradiology University Medicine Greifswald Greifswald Germany; ^3^ Department of Radiology University of Wisconsin Madison Wisconsin USA

**Keywords:** developmental dysplasia of the hip, lumbopelvic alignment, pelvic tilt, periacetabular osteotomy, sex

## Abstract

**Purpose:**

The functional hip–spine interaction is increasingly noted in hip preservation by periacetabular osteotomy (PAO), while potentially affecting the impingement‐free acetabular reorientation. However, the clinically relevant sex‐related differences in lumbopelvic alignment have been poorly studied. Thus, the purpose of this study was to evaluate a matched PAO patient cohort for sex‐related differences in lumbopelvic alignment.

**Methods:**

Out of 138 patients undergoing PAO between January 2024 and September 2024 at one high‐volume centre, there were 68 data sets (34 male, 34 female) included. The data sets of this diagnostic cohort study were prospectively collected, and the patients were matched in a 1:1 ratio for sex, age and acetabular morphology (hip dysplasia, borderline hip dysplasia, acetabular retroversion). Lumbopelvic alignment was assessed with serial sagittal lumbopelvic radiographs in standing, relaxed‐seated and deep‐seated positions. Each radiograph was reviewed for pelvic tilt (PT), lumbar lordosis and sacral slope.

**Results:**

Females showed a significantly lower PT in standing (7.8 vs. 14.3°, *p* < 0.001), relaxed‐seated (28.1 vs. 34.9°, *p* = 0.012) and deep‐seated (3.7 vs. 11.0°, *p* = 0.013) positions. Furthermore, females had a significantly increased lumbar mobility (Δ relaxed‐seated − deep‐seated position − 35.4° vs. 27.0°, *p* = 0.003), while there was no sex‐related difference in sacral mobility (*p* > 0.05).

**Conclusion:**

There are sex‐related differences in functional lumbopelvic alignment across various positions of daily living in patients undergoing PAO. With a greater anterior PT, females are at risk of an anterior hip impingement. Thus, the intraoperative anterior and posterior wall reorientation by PAO should be adapted to the sex‐related lumbopelvic alignment to ensure an impingement‐free surgical outcome.

**Level of Evidence:**

Level IV, case series.

**Abbreviations**
AI: acetabular inclination
AR: acetabular retroversion
BHD: borderline hip dysplasia
HD: hip dysplasia
LCEA: lateral centre‐edge angle
LL: lumbar lordosis
PAO: periacetabular osteotomy
PT: pelvic tilt
SS: sacral slope
THA: total hip arthroplasty
Δ: delta

## INTRODUCTION

The functional lumbopelvic relation, initially studied in patients undergoing total hip arthroplasty (THA), has gained attention even in hip preservation surgery by periacetabular osteotomy (PAO) [3, 5]. PAO aims for a three‐dimensional acetabular reorientation, which is potentially affected by the individual functional lumbopelvic alignment [9].

Regarding the dynamic interaction between the spine, pelvis and femur, the acetabular orientation and femoral head coverage differ significantly in daily activity between supine, seated and standing posture [13, 18]. Additionally, data on typical acetabular morphologies in PAO highlighted the differences in pelvic tilt (PT) between hip dysplasia (HD) and acetabular retroversion (AR) [7, 12, 25]. Thus, a thorough understanding of the patient–individual lumbopelvic interaction is needed to achieve an impingement‐free range of motion postoperatively [29].

However, the sex‐related functional lumbopelvic alignment in patients undergoing PAO has not yet been studied. In general, sex‐specific characteristics in orthopaedic studies are rarely investigated and studies on hip preservation by PAO are mainly focused on females [10, 11, 16, 26]. Even recent studies on pelvic and acetabular morphology in PAO patients were not balanced for sex with a relevant overhang of female patients [6, 8]. However, precise patient‐individual acetabular reorientation is essential for achieving successful clinical outcomes, with overcorrection in the coronal plane already identified as a risk factor for PAO failure [1, 6]. In this respect, acetabular anteversion in the transverse plane showed a strong association with PT [18]. Thus, sex‐related differences in functional lumbopelvic alignment may necessitate individualized target zones for acetabular reorientation in males and females.

Therefore, comprehensive preoperative diagnostics should consider the patient's individual lumbopelvic alignment to achieve a precise three‐dimensional acetabular reorientation.

Consequently, a prospective, single‐centre diagnostic matched‐cohort study was initiated to investigate the sex‐related functional lumbopelvic alignment in a representative PAO patient cohort. The combination of a matched‐cohort balanced for sex, age and acetabular morphology with a consecutive series of sagittal lumbopelvic radiographs in standing, relaxed‐seated and deep‐seated positions aimed to improve the lumbopelvic understanding in patients undergoing PAO. We hypothesized that there is a relevant sex‐related difference in functional lumbopelvic alignment in patients undergoing PAO.

## MATERIAL AND METHODS

### Patient cohort and study design

**Figure 1:**
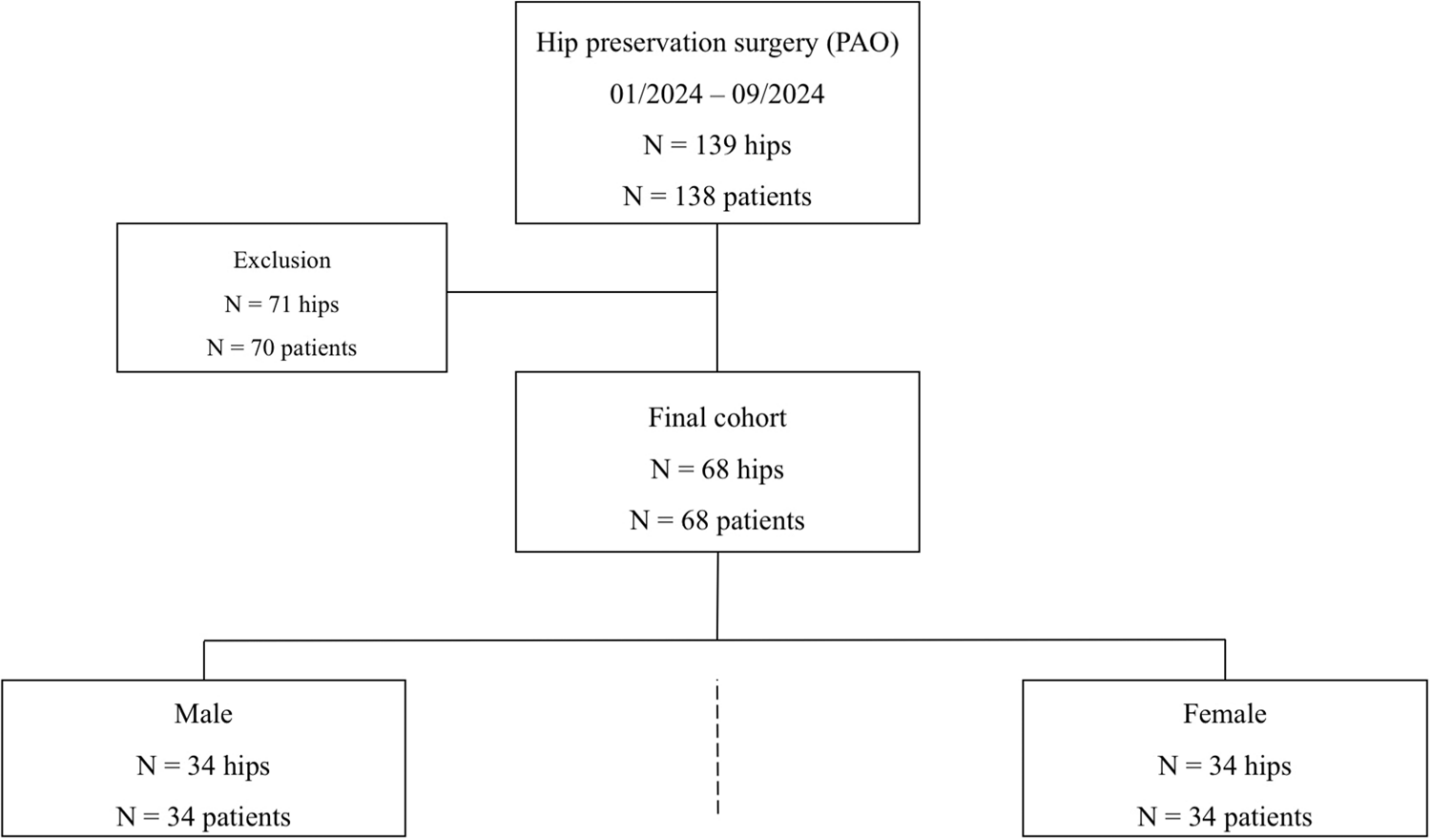
Flowchart after matching for sex, age and acetabular morphology. PAO, periacetabular osteotomy.

Patients undergoing hip preserving surgery via PAO for symptomatic HD, borderline HD (BHD) and AR, older than 18 years of age, were prospectively enrolled in this review board‐approved, diagnostic cohort study (Figure 1).

Patients presented for PAO at one high‐volume centre for hip preservation surgery due to refractory hip pain (>6 months) and failure of conservative therapy. The treatment decision was made based on a combination of patient‐reported symptoms, physical examination and radiographic parameters. Radiographically, indications for PAO were no or only mild signs of osteoarthritis (Tönnis grade <2), a congruent hip joint and a lateral centre‐edge angle (LCEA) < 25° or presence of AR. All patients gave written informed consent prior to study enrolment.

The sample size was calculated to have a power of 0.80 (1 − β). Thirty‐four patients were required in each group. To enhance the comparability of the study groups, a matched‐pair analysis was performed. The patients were matched in a 1:1 ratio for sex, age and hip deformity (classified as HD, BHD, AR) (Table 1).

**Table 1:** Patient characteristics and radiographic acetabular morphology after matching for sex, age and acetabular morphology.

	Male (*N* = 34)	Female (*N* = 34)
Mean age, (years)	28.97	28.98
HD (*n*)	12	12
Mean LCEA (°)	11.41	13.00
Mean AI (°)	19.92	14.92
BHD (n)	15	15
Mean LCEA (°)	21.66	21.06
Mean AI (°)	8.73	10.93
AR (*n*)	7	7
Mean LCEA (°)	32.86	33.14
Mean AI (°)	−1.71	0.14

Abbreviations: AI, acetabular inclination; AR, acetabular retroversion; BHD, borderline hip dysplasia; HD, hip dysplasia; LCEA, lateral centre‐edge angle.

Age matching was conducted with a permissible deviation of up to 5% to ensure a balanced age distribution. Out of 138 patients, the final analysis included the complete data sets of 68 patients (Table 1). 70 patients were excluded in accordance with the calculated sample size (Supporting Information S1: Table 1).

### Radiographic assessment

Each patient underwent a consecutive series of sagittal lumbopelvic radiographs in standing, relaxed‐seated (hip flexion 90°, femurs parallel to the floor) and deep‐seated positions (maximal forward‐leaning, femurs parallel to the floor). The field of view included the lumbar spine, pelvis and femoral heads. Regarding the enlarged field of view, the source‐to‐detector distance was elevated to 180 cm. Examinations were performed on a Philips DigitalDiagnost C90 (Koninklijke Philips N.V.). With respect to the young age of our patient population, an adapted low‐dose radiation protocol was introduced by halving the shutoff dose, applying an additional 0,1 mm Cu to the 1 mm Al filter and increasing the tube voltage from 77 to higher kV dependent on the patient's BMI. For a normal BMI patient, tube voltage was increased from 77 to 99 kV; consequently, the current‐time product decreased from 99,832 to 33,564 mAs. Finally, the fluoroscopy area dose product decreased from 89 to 53 cGy × cm².

**Figure 2:**
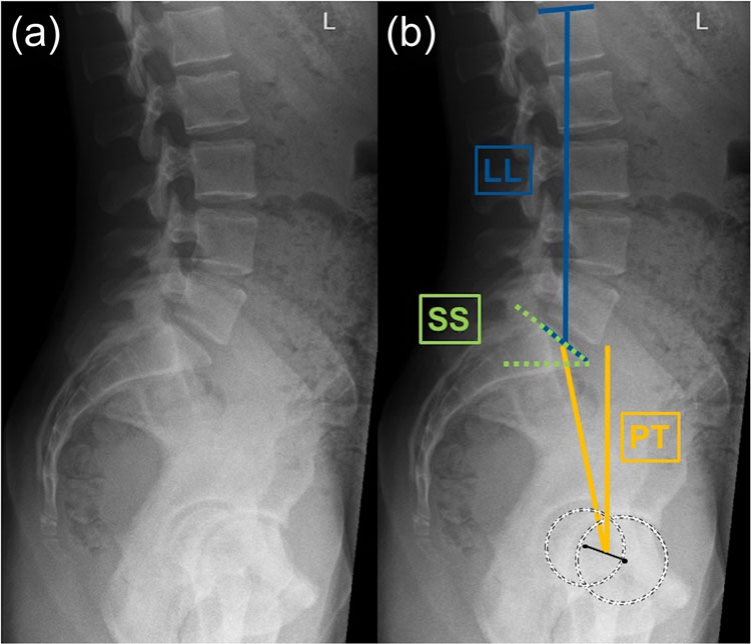
Representative case illustrating the lumbopelvic measurement. (a) Native standing lumbopelvic radiograph. (b) Measurement of pelvic tilt (PT) (yellow), lumbar lordosis (LL) (blue) and sacral slope (SS) (green).

The radiographs were reviewed by the first author (M. F.) (orthopaedic surgeon) for PT, lumbar lordosis (LL) and sacral slope (SS) (Figure 2), and the lumbopelvic mobility (lumbar mobility and sacral mobility) was defined as the difference (Δ value) between the above‐mentioned positions. For validation, 25% of included patients were assessed by two additional raters (orthopaedic surgeon and musculoskeletal radiologist) to ensure high measurement quality. The intraclass correlation coefficient was 0.997 for PT, 0.968 for LL and 0.987 for SS.

The lumbopelvic variables were measured as follows (Figure 2):

PT—Angle between the vertical axis and a line connecting the centre of the S1 endplate and the femoral head centre.

LL—Angle between L1 and S1 endplates.

SS—Angle between S1 endplate and a horizontal reference line.

Acetabular morphology was reviewed on standardized anteroposterior pelvic radiographs. HD was classified using an LCEA cutoff value of <18°, while BHD was defined through an LCEA between 18° and 25°. AR was defined by the presence of all three signs of AR (crossing over, posterior wall and sciatic spine sign) on anteroposterior pelvic radiographs, combined with an LCEA > 25°, as previously described (Table 1) [19].

### Data analyses

Descriptive statistics were used to summarize the patient characteristics and radiographic measurements. Statistical analyses were performed using SPSS (version 29, IBM). The sample size was calculated to have a power of 0.80 (1 − β). Thirty‐four patients were required in each group. G*Power software was used to calculate the sample size (version 3.1.9.4). Differences between the groups were tested for statistical significance using the nonparametric *Mann–Whitney U‐test*. A *p *< 0.05 was considered statistically significant.

## RESULTS

**Figure 3:**
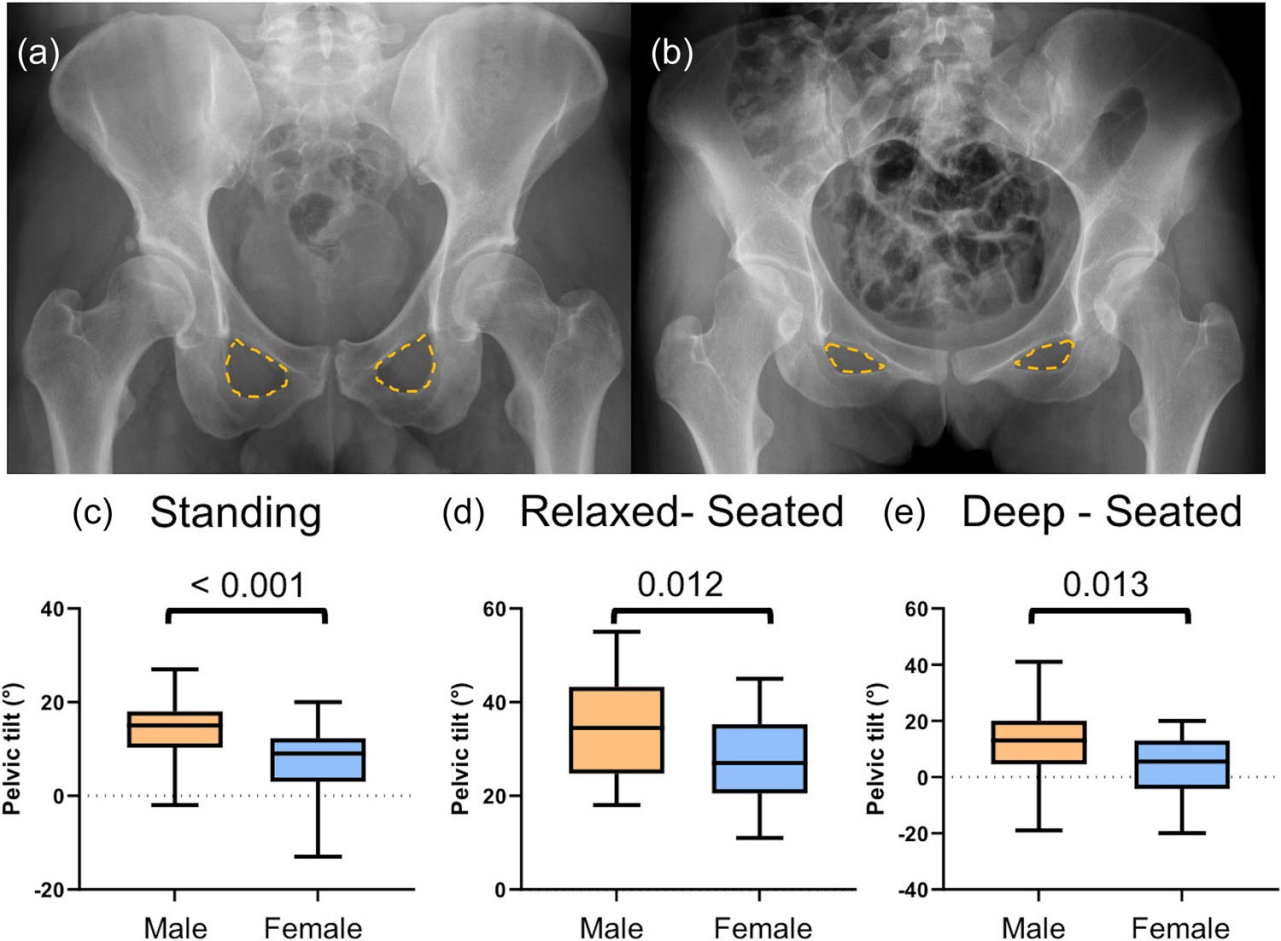
Sex‐related difference in PT. (a and b) The obturator foramen projection (yellow line) is the first indicator for differences in PT between males (a) and females (b) on anteroposterior pelvic radiographs. (c–e) Sagittal PT in standing, relaxed‐seated and deep‐seated positions. Mann–Whitney U‐test, *p* < 0.05.

The PT was significantly lower in standing (7.8° vs. 14.3°, *p *< 0.001), relaxed‐seated (28.1° vs. 34.9°, *p *= 0.012) and deep‐seated (3.7° vs. 11.0°, *p *= 0.013) positions in females (Figure 3).

Comparing the static lumbopelvic alignment between both sexes, there was a trend to reduced LL in female patients when bending maximal forward (deep‐seated position), while not reaching statistical significance (−4.9 vs. −0.1°, *p *= 0.181) (Table 2). The SS showed no statistical sex‐related difference in all analyzed positions of daily living (standing—*p *= 0.415, relaxed‐seated—*p *= 0.309, deep‐seated—*p *= 0.405) (Table 2).

**Table 2:** Static lumbopelvic parameters and lumbopelvic mobility (Δ value) in standing, relaxed‐seated and deep‐seated positions.

	Male (*N* = 34)	Female (*N* = 34)	*p* Value
Lumbar lordosis—Standing (°), mean (range)	58.2 (27–74)	57.7 (22–76)	*0.805*
Lumbar lordosis—Relaxed seated (°), mean (range)	26.9 (1–53)	30.5 (3–56)	*0.289*
Lumbar lordosis—Deep seated (°), mean (range)	−0,1 (−17 to 24)	−4,9 (−31 to 18)	*0.181*
Δ Lumbar lordosis ‐standing—Relaxed seated (°), mean (range)	31.2 (9–60)	27.1 (5–53)	*0.217*
**Δ Lumbar lordosis relaxed seated—Deep ‐seated (°), mean (range)**	**27.0 (8–49)**	**35.4 (12–57)**	***0.003***
Sacral slope—Standing (°), mean (range)	37.8 (18–56)	39.8 (20–74)	*0.415*
Sacral slope—Relaxed ‐seated (°), mean (range)	19.1 (4–32)	21.5 (−6 to 48)	*0.309*
Sacral slope—Deep seated (°), mean (range)	43.2 (16–69)	45.9 (18–72)	*0.405*
Δ Sacral slope standing—Relaxed seated (°), mean (range)	18.7 (4 –7)	18.3 (1–41)	*0.791*
Δ Sacral slope relaxed seated—Deep ‐seated (°), mean (range)	22.9 (−43 to 22)	24.4 (−48 to −3)	*0.953*

*Note*: Mann–Whitney U‐test, *p *< 0.05. Bold values indicate significant intergroup difference. Mann‐Whitney U‐test, *p* < 0.05.

Lumbar mobility was significantly greater in females (Δ relaxed‐seated − deep‐seated—*p *= 0.003) (Table 2). Comparing the sex‐related sacral mobility, there was no significant difference between male and female patients (Table 2).

## DISCUSSION

The main findings of this study were (I) females undergoing PAO exhibited a more anteriorly tilted pelvis across all analyzed positions of daily living and (II) females had a greater lumbar mobility toward positions of peak motion compared to males. Therefore, intraoperative acetabular reorientation in PAO should consider a sex‐related adjustment in anterior and posterior wall positioning, as greater anterior wall coverage in females may cause impingement due to an anterior tilted pelvis.

The current study found that females undergoing PAO had a greater anterior PT compared to their male counterparts. Even though this study is the first to illustrate a sex‐related difference in sagittal PT in patients undergoing PAO, this is in line with data on females under 50 years of age undergoing THA. Cha et al. found in their study on patients with secondary osteoarthritis due to HD that the anterior PT in females remains unchanged as osteoarthritis progresses, indicating a constant difference in sex‐related lumbopelvic alignment [4]. Therefore, the anterior PT in females should be considered intraoperatively in general for both—hip preservation by PAO and THA.

Besides the patients' sex, the PT was already associated with acetabular metrics in dysplastic as well as impingement‐driven hip pathologies [12, 15]. Recently, Haertlé et al. described the PT as a component of the HD phenotype, noting differences even between anterolateral and posterolateral dysplasia [12].

In the context of the current literature and the results of this study, there is increasing evidence for a complex interplay between patient individual factors, acetabular morphology and PT in patients with prearthritic hip disease. Furthermore, the findings of our study illuminate a potential clinical predisposition of females for an anterior joint impingement caused by the more anterior tilted pelvis. Consequently, our data emphasize the extension of preoperative diagnostics to include a lumbopelvic evaluation before planning acetabular reorientation via PAO.

The current study is the first focusing on a symptomatic patient cohort undergoing PAO and reporting sex‐related differences in sagittal lumbopelvic alignment. In contrast to this data, Mizukoshi et al. found no differences in PT between asymptomatic males and females in standing and relaxed‐seated positions. However, similar to the current study results, increased lumbar mobility was found in asymptomatic females of their cohort [21]. One potential reason for these conflicting results compared to the current symptomatic PAO cohort may be the sex‐related differences in the severity of symptoms at the time of surgery [23]. It has been shown that females frequently undergo surgical treatment at a later stage of disease, which is worth noting in the field of elective surgery [2, 14, 17]. In line with this data, poor preoperative patient‐reported outcomes of females for pain, daily activity and joint functionality have been reported for several orthopaedic interventions [20, 22].

Even when the current study did not focus on patient‐reported outcome parameters, the findings of this diagnostic cohort study are of interest for further therapeutic adjustments. In this regard, PAO has several similarities to THA procedures, including challenges in achieving optimal outcomes when cup positioning or acetabular reorientation is inadequate [1, 6, 24, 27]. The data of this matched cohort study indicate that PAO in females should consider an adapted anterior wall reorientation to reduce the risk of a secondary impingement caused by an anterior tilted pelvis. The clinical relevance is further underscored by data from Tateishi et al. describing the clinical Hip–Lumbar Mobility test (HLMT) for the preoperative clinical spinopelvic assessment in patients undergoing hip preserving surgery. In their study, HLMT‐positive patients achieved significantly lower postoperative outcomes [28]. These findings highlight the importance of evaluating sagittal lumbopelvic alignment preoperatively, providing a useful combination of a clinical test and the current radiographic assessment of functional lumbopelvic alignment.

Nevertheless, this study has several limitations. Although the current study reported on a representative study cohort with various prearthritic hip deformities, the findings may not be generalizable to other populations, as the study was conducted at a single university centre with a specific patient selection. Furthermore, this study focuses on an osteotomy cohort, which limits its generalizability to the overall hip preservation population including arthroscopic‐treated patients. However, it is important to note that the current study cohort was matched for sex, age and acetabular morphology, which strengthens the validity of the results and reduces the risk of underpowering the findings in males, frequently observed in PAO studies. Next, this study only included lumbopelvic and acetabular metrics, without assessing femoral deformities contributing to the functional lumbopelvic interaction. Additionally, the results remain at risk for an individual performance bias, especially in positions of peak motion. Due to the diagnostic focus of this study, patient‐reported outcome parameters were not included in this analysis.

## CONCLUSION

The anterior PT in females increases the risk of anterior joint impingement, and the intraoperative acetabular reorientation should avoid excessive anterior wall reorientation in females. Future studies should focus on sex‐related PAO outcomes, considering lumbopelvic‐adjusted treatment protocols.

## AUTHOR CONTRIBUTIONS

**Maximilian Fischer**: Conceptualization; methodology; formal analysis and investigation; visualization; writing—original draft preparation; writing—review and editing. **Lars Nonnenmacher**: Conceptualization; formal analysis and investigation; writing—review and editing. **Matthias R. Mühler**: Conceptualization; methodology; writing—review and editing. **Georgi I. Wassilew**: Conceptualization; methodology; writing—review and editing. **Andreas Nitsch**: Formal analysis and investigation; writing—review and editing. **Andre Hofer**: Writing—review and editing.

## CONFLICT OF INTEREST STATEMENT

Georgi I. Wassilew has received research support from Enovis and Smith & Nephew without relevance to the content of this article. The remaining authors declare no conflict of interest.

## ETHICS STATEMENT

Institutional review board approval (BB099/20a) was obtained from the local independent ethics committee of the University Medicine Greifswald according to the World Medical Association Declaration of Helsinki. All patients gave written informed consent prior to inclusion.

## Supporting information

Supporting information.

## Data Availability

The data that support the findings of this study are available from the corresponding author upon reasonable request.
