# Supplementary material for: Greater anterior pelvic tilt and lumbar mobility in females compared to males undergoing periacetabular osteotomy: A matched cohort study
Source: J Exp Orthop. 2025 Feb 10;12(1):e70167. doi: 10.1002/jeo2.70167 (PMC11808264; doi:10.1002/jeo2.70167)
Supplement: Supplementary file 1 — Supporting information. [file JEO2-12-e70167-s001.docx]

|  | Excluded patients  (N = 70) |
| --- | --- |
| Mean age, (years) | 32,56 |
| HD (n)  *Mean LCEA (°)*  *Mean AI (°)* | 46  *10,35*  *18,02* |
| BHD (n)  *Mean LCEA (°)*  *Mean AI (°)* | 21  *20,33*  *10,42* |
| AR (n)  *Mean LCEA (°)*  *Mean AI (°)* | 3  *28*  *0* |

Supplementary Table 1. Patient characteristics and radiographic acetabular morphology of the excluded patients. HD hip dysplasia, BHD borderline hip dysplasia, AR acetabular retroversion, AI acetabular inclination, LCEA lateral center-edge angle
